# Supplementary material for: Resolving the full spectrum of human genome variation using Linked-Reads
Source: Genome Res. 2019 Apr;29(4):635–45. doi: 10.1101/gr.234443.118 (PMC6442396; doi:10.1101/gr.234443.118)
Supplement: Supplemental Material [file supp_gr.234443.118_Supplemental_longranger-2.2.zip › longranger-2.2.tar.bz2!/lr2.2-public/lib/stan/cmdstan/stan/doc/api/html/promote__common_8hpp_source.html]

Stan: src/stan/math/matrix/promote\_common.hpp Source File


|  |  |
| --- | --- |
|  | Stan  2.5.0  probability, sampling & optimization |


- Main Page
- Related Pages
- Namespaces
- Classes
- Files

- File List
- File Members

All Classes Namespaces Files Functions Variables Typedefs Enumerations Enumerator Friends Macros Pages

- src
- stan
- math
- matrix

promote\_common.hpp

Go to the documentation of this file.

1 #ifndef STAN\_\_MATH\_\_MATRIX\_\_PROMOTE\_COMMON\_HPP

2 #define STAN\_\_MATH\_\_MATRIX\_\_PROMOTE\_COMMON\_HPP

3

4 #include <stan/math/matrix/common\_type.hpp>

5 #include <stan/math/matrix/promoter.hpp>

6

7 namespace stan {

8

9  namespace math {

10

11  template <typename T1, typename T2, typename F>

12  inline

13  typename common\_type<T1,T2>::type

14  promote\_common(const F& u) {

15  return promoter<F, typename common\_type<T1,T2>::type>

16  ::promote\_to(u);

17  }

18

19  }

20 }

21

22

23 #endif

promoter.hpp

common\_type.hpp

stan::math::promoter

**Definition:** promoter.hpp:14

stan::math::promote\_common

common\_type< T1, T2 >::type promote\_common(const F &u)

**Definition:** promote\_common.hpp:14


---

[ Stan Home Page ]

*© 2011–2014,
Stan Development Team.*
